# Supplementary figures and images for: The Impact of BMI Changes on the Incidence of Glomerular Hematuria in Korean Adults: A Retrospective Study Based on the NHIS-HEALS Cohort
Source: Biomedicines. 2023 Mar 22;11(3):989. doi: 10.3390/biomedicines11030989 (PMC10046077; doi:10.3390/biomedicines11030989)

Supplementary Figure S1.

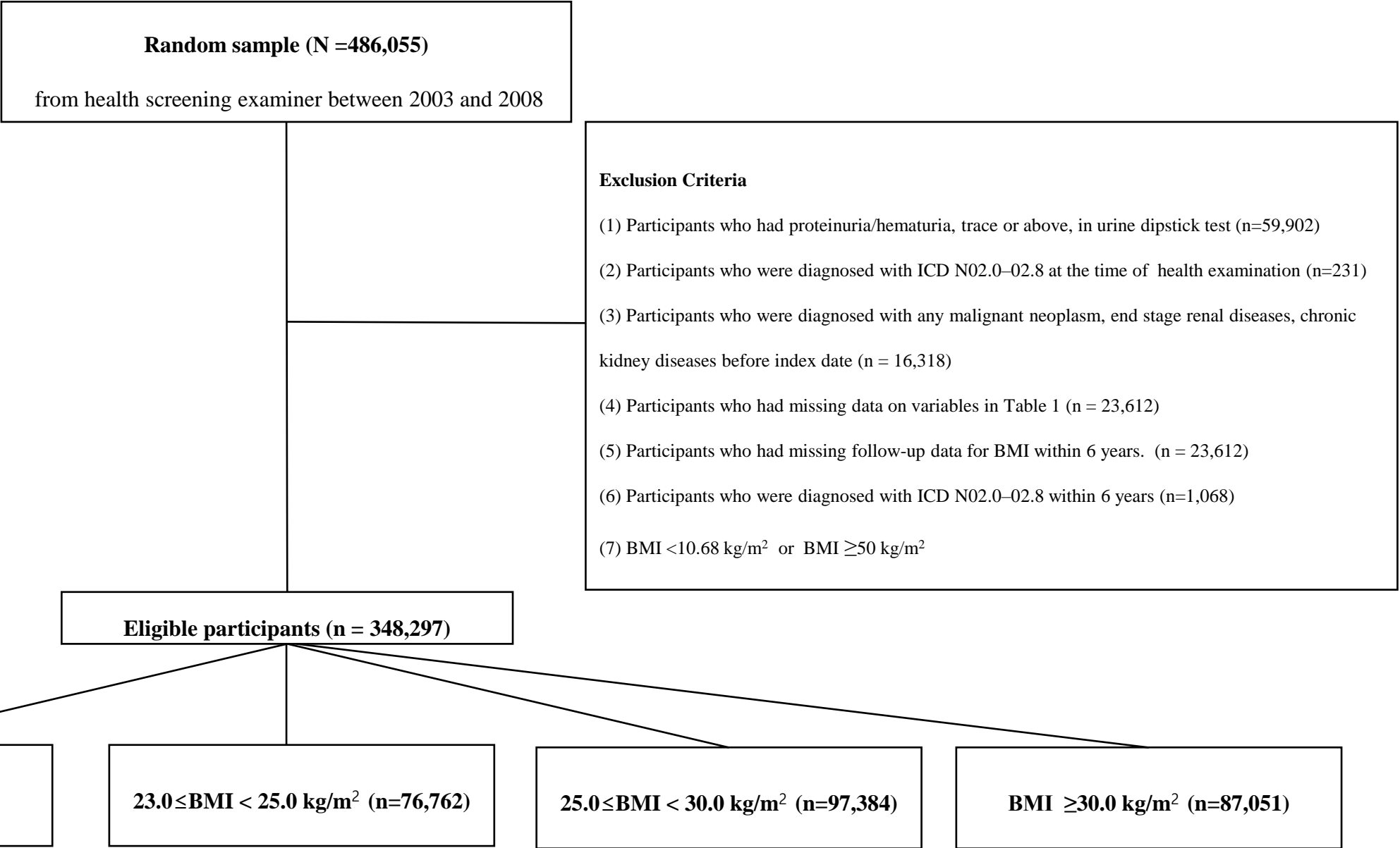

Supplement: Supplementary file 1 [file biomedicines-11-00989-s001.zip › biomedicines-2287676-supplementary.pdf]
